# Meetings of Societies

**Published:** 1909-03

**Authors:** 


### MEETINGS OF SOCIETIES.

### Bristol Medico-Chirurgical Society.

*December 9th*, 1908.

Dr. MICHELL CLARKE, President, in the Chair.

Mr. A. L. FLEMMING read a paper on **Post-anæsthetic vomiting.** (See page 40.)

Mr. HEY GROVES read a paper on **Three Cases of Ileo-Colostomy,** in which he had performed the operation (i) for chronic mechanical obstruction, (ii) for tuberculous perforation of the cæcum, (iii) for idiopathic dilatation and atony of the colon. In the latter case excision of practically the whole colon was subsequently performed with satisfactory result, the patient being a neurasthenic, middle-aged woman. He was persuaded, from the success in these cases, that the operation was at times of great value, although occasionally the anti-peristaltic wave, which normally occurs in the ascending and transverse colon, gave rise to painful distension in the occluded portion of the bowel.

Dr. ELWIN HARRIS related the case of a woman, aged 40, the subject of obstinate constipation, who had already undergone Weir Mitchell treatment and an operation for nephropexy. He performed a right inguinal colotomy with a view to irrigation of the lower bowel, whereby she was temporarily relieved. But a sudden acute abdominal attack led to a further exploratory laparotomy, revealing a thickened, inflamed U-shaped transverse colon ; nothing more was done at the time. Fæcal impaction recurred, and ileo-sigmoidostomy was performed. At first natural actions of the bowel followed, but there was no permanent improvement, and after two months fæces were again passed through the colotomy wound. Four months afterwards the greater part of the large intestine was removed, but the patient died on the second day after the operation. He was impressed by the hopelessness of permanently relieving by any operation the protean complaints of a neurasthenic.

Mr. CARWARDINE showed lantern slides prepared from **a Case of early Pregnancy** with photo-micrographs of the fœtal villi and syncytium.

Dr. WALTER SWAYNE said that the earliest human ovum studied hitherto was about the eleventh day (Bryce), and it had served to demonstrate the fictitious character of a human embryology based chiefly on the development of invertebrates.

It was interesting in connection with this case to note that one of the first specimens which upset the old idea of the formation of the decidua reflex was obtained from a case of tubal pregnancy (para-tubal hæmatocele). The tubal mucosa showed a distinct scar at the point where the ovum had penetrated the wall of the Fallopian tube, owing to the "boring" action of the trophoblast.

Dr. Stack read a paper on **Subcutaneous Injection of Paraffin,** showing several cases of restoration of the bridge of the nose.

Dr. W. Kenneth Wills read a short note on an **urticarial rash occurring among grain-porters employed in unloading barley from an African port.** Fine vegetable hairs were found in the papules which resembled somewhat those of the "Mucuna" (cowhage), though with some microscopical differences. The rash was of a very acute nature, but seemed to yield to inunctions of a chrysarobin ointment.

---

*January* 13*th*, 1909.

Dr. Michell Clarke, President, in the Chair.

Dr. Charles read notes of a case of **Fœtal Rickets** (case shown).

Dr. Edgeworth read a paper on the **Temporary Paralyses which may occur in cases of Arterio-sclerosis.**

Dr. Parker read a paper on the **Causes of Transient Cerebral Paralyses.** (See p. 15.)

Dr. Bertram Rogers read a paper on **Physique and our Schoolboys.** (See p. 27.)

---

*February* 10*th*, 1909.

Dr. Michell Clarke, President, in the Chair.

The evening was devoted to a discussion on **Milk,** and the following papers were read:—

**Milk from the Public Health Standpoint,** by Dr. D. S. Davies.

**The Adulteration of Milk,** introduced by Dr. E. Russell.

**Some Impurities of Bristol Milk, based upon the Examination of 240 Specimens,** by Dr. Walker Hall.

**The Milk Curdling and other Activities of the Commercial Preparations of Lactic Acid Ferments,** by Dr. Walker Hall and Dr. W. A. Smith.

**Contra-indications to the use of Milk in Disease,** by Dr. Michell Clarke.

**Infantile Diseases and Milk Diet,** by Dr. Bertram Rogers.

**The Heating of Milk as it affects the Nutrition and Health of the Infant,** by Dr. Fortescue-Brickdale.

For an abstract of these papers see previous pages of the *Journal*.

J. Lacy Firth,
J. A. Nixon, *Hon. Sec.*

---

